# Mechanism of Traditional Tibetan Medicine Grubthobrildkr Alleviated Gastric Ulcer Induced by Acute Systemic Hypoxia in Rats

**DOI:** 10.1155/2022/4803956

**Published:** 2022-04-05

**Authors:** Mei Yang, Zhanting Yang, Yongfang Li, Shanshan Su, Zhanqiang Li, Dianxiang Lu

**Affiliations:** ^1^Medical College of Qinghai University, Xining 810001, China; ^2^Research Center for High Altitude Medicine, Key Laboratory of Application and Foundation for High Altitude Medicine Research in Qinghai Province (Qinghai-Utah Joint Research Key Lab for High Altitude Medicine), Qinghai University, Xining 810001, China; ^3^Xining Customs District, Key Laboratory of Food Safety Research in Qinghai Province, Xining 810003, China

## Abstract

**Objective:**

This study was aimed at investigating the potential mechanism of Grubthobrildkr (GTB) on systemic hypoxia-induced gastric ulcers in rats and at detecting the chemical profile of GTB.

**Methods:**

Male Sprague-Dawley rats were separated into control, hypoxia, hypoxia+omeprazole, and hypoxia+GTBs (0.25, 0.5, and 1.0 g·kg^−1^·d^−1^) groups. Systemic hypoxia was created in a hypobaric chamber to simulate 5000 m high altitude by adjusting the inner pressure and oxygen content for 6 days. After that, the ulcer index, pH, and volume of gastric juice were assessed. The levels of endothelin-1 (ET-1), gastrin (GAS), motilin (MTL), phospholipase A_2_ (PLA_2_), and prostaglandin E_2_ (PGE_2_) were detected by ELISA. The expression level of hydrogen potassium ATPase (H^+^-K^+^-ATPase), cyclooxygenase-1 (COX-1), and cyclooxygenase-2 (COX-2) was tested by western blotting. Chemical profile of GTB was revealed by UHPLC-Q-exactive hybrid quadrupole-orbitrap mass (UHPLC-Q-Orbitrap MS).

**Results:**

GTB decreased the ulcer index in rats under hypoxia for six days, which was related to increased pH and volume of gastric juice, enhanced MTL and PGE_2_ levels, and decreased ET-1 and PLA_2_ levels of gastric mucosa. Furthermore, GTB decreased the level of H^+^-K^+^-ATPase and COX-2 while increased COX-1 levels in gastric mucosal tissue. 44 constituents were identified by UHPLC-Q-Orbitrap MS in GTB.

**Conclusion:**

GTB exerted a gastroprotective effect to alleviate gastric ulceration induced by acute systemic hypoxia in rats. The effect of GTB increasing the volume and pH of gastric juice in rats under acute systemic hypoxia could be regulated by gastrointestinal hormones, including MTL and ET-1. Mechanically, gastrointestinal protection of GTB was based on inhibition of the protons pumping H^+^-K^+^-ATPase and regulation of prostaglandin family in rats.

## 1. Introduction

Some symptoms of digestive system such as peptic ulcer were frequently found in mountaineers and altitude people [1]. Both gastric acid and mucosal ischemia were involved in the etiology of stress ulcers [2]. In general, a physiological balance was maintained between gastric acid secretion and gastric mucosal defense. Mucosal lesions and subsequent gastric ulcers appeared when the balance was disrupted. The decrease in gastric mucosal protective mechanism can be induced by many factors, including hypoxia [3]. A decrease in gastric mucosal blood flow led to gastric ischemia by destroying the lining of the mucosa, which is closely related to systemic hypoxia. The secretion of gastric acid is regulated by various gastrointestinal hormones, such as gastrin (GAS), motilin (MTL), and endothelin (ET) [4]. These gastrointestinal hormones also influenced the level of intercellular Ca^2+^ and eventually activated H^+^-K^+^-ATPase. An inhibition of protons pumping H^+^-K^+^-ATPase as a means of preventing gastric ulcer has attracted considerable attention for several years [5].

Prostaglandins (PGs) were a family of lipid compounds derived from the arachidonic acid pathway and mediated several physiological functions, including the regulation of inflammation and gastrointestinal protection [6]. PGs were not only found to prevent the formation of ulcers but also improve the healing of the ulcer [7]. According to the reports, the secretion of gastric acid was regulated by PGs, which increased mucosal blood flow and promoted the healing of the mucosa. Enzymes involved in PG synthesis include PLA_2_, which influences the production of arachidonic acid, COX-1, and COX-2. The restoration of PGE_2_ to normal levels can reduce gastric mucosa lesions [8, 9].

Traditional Tibetan medicine is commonly used in Qinghai and Tibetan folk medicine to treat several gastric problems [10]. Grubthobrildkr (GTB), a Tibetan traditional medicine formula, composed of seven medicine components, *Gypsum Calcitumrubrum*, *Calcite*, *Corydalis hendersonii* Hemsl, *Terminalia chebula* Retz (enucleation), *Radix aucklandiae*, *Faeces Trogopterori*, *Apis cerana Fabr*, and *Lagotis brevitub* Maxim at a ratio of 4 : 2.4 : 3.6 : 2.4 : 2 : 1 : 2.4, had been widely used in ethnomedicine for the clinical therapy of gastrointestinal diseases [11, 12].

In our previous study, we established a systemic hypoxia-induced gastric ulcer rat model by feeding rats in hypobaric chamber-stimulated altitude of 5000 m for 2, 4, 6, 8, and 10 days, respectively, and the severe gastric ulcer was found in the 6-day hypoxia group [13]. We also found the protective effect of GTB on systemic hypoxia-induced gastric ulcers in rat [14]. However, it remains to elucidate the mechanism of GTB on stress ulcer induced by systemic hypoxia. In this article, we focused on detecting the gastrointestinal protective mechanism of GTB in rats.

## 2. Experimental

### 2.1. Medicine Material and Preparation

GTB was purchased from Qinghai Provincial Tibetan Medical Hospital, the authority in the area on Tibetan medicine, with the batch number of Z20110562. According to the specification, the recommended dosage of GTB for adults was 3.0 g (total raw materials/day). In rat, equivalent dose was about 7 times the human dose. Based on clinical observation of the safety of this medicine, we chose 5, 10, and 20 times the human dose as lower (0.25 g·kg^−1^·d^−1^), middle (0.5 g·kg^−1^·d^−1^), and high dosage (1.0 g·kg^−1^·d^−1^), respectively. Three doses of GTB were suspended in distilled water and administrated by oral gavage for 6 days in this study. Omeprazole (Zhejiang Bohua Chemical Co., Ltd. Batch No. 1410021) at a dosage of 7 mg·kg^−1^·d^−1^ was used as a positive control medicine. Omeprazole was similarly suspended in distilled water and was mixed vigorously before oral gavage administration.

### 2.2. Animal

The study was approved by the Institutional Animal Care and Use Committee of the Qinghai University in accordance with NIH guidelines for the care and use of laboratory animals. Male Sprague-Dawley rats (220–240 g) were obtained from Gansu Traditional Chinese Medicine College, China (certificate of quality: SYXK (甘) 2011-0001). The rats were housed with a 12 h light-dark cycle at 25°C ± 2°C and in a relative humidity of 50%–60%. The rats were fed *ad libitum* on a diet of standard pellets and water. All possible efforts were made to minimize suffering and reduce the number of rats used. No rat died during the experiment. Sprague-Dawley rats were randomly divided into control, hypoxia, hypoxia+omeprazole, and hypoxia+GTBs (0.25, 0.5, and 1.0 g·kg^−1^·d^−1^) groups, with each group comprising of 12 rats. The hypoxic groups were exposed in hypobaric chamber (Guizhou Fenglei Aviation Ordnance Co., Ltd. DYC-3000), equal to the parameter in altitude of 5000 m. The rats were deprived of food for 24 h before research time point. Finally, the rats were sacrificed by bleeding from the abdominal aorta under urethane anesthesia (1.0 g·kg^−1^).

### 2.3. Measurement of pH, Volume of Gastric Juice, and Ulcer Index in Gastric Ulcer Tissue

The gastric secretion from sacrificed rat was gathered. The gastric content was centrifuged at 3000 rpm for 20 min (4°C), the volume of the gastric juice appearing in the supernatant was determined, and the total acidity was tested by pH 211 meter (Mettler Toledo Company). For ulcer index measurement, the stomach of the rat in each group was immediately filled with 5 mL of 10% phosphate-buffered formalin (pH 7.0) and submerged in the same solution for 30 min. To evaluate the extent of damage, the gastric sections were opened along the greater curvature and rinsed with normal saline to remove gastric content and blood clot. The degree of gastric mucosal damage was evaluated and rated for gross pathology according to the ulcer score scale described by Dekanski et al. [15] The criteria for assessing macroscopic damage were scored as follows: no ulcer (score = 0), ulcer < 1 mm (score = 1), 1 < ulcer < 2 mm (score = 2), 2 < ulcer < 3 mm (score = 3), and 3 < ulcer < 4 mm (score = 4). The sum of the total score was divided by the number of rats to obtain mean ulcer index for each group. The inhibition percentage was calculated using the following formula: [(UI untreated − UI treated)/UI untreated] × 100.

### 2.4. Determination of ET-1, GAS, and MTL Level in Blood

Enzyme-linked immunosorbent assay (ELISA) kits were utilized to measure serumal ET-1, GAS, and plasmic MTL level. The test was performed in accordance with reagent instructions. The kits were obtained from R&D Systems, USA.

### 2.5. Determination of PLA_2_ and PGE_2_ Level in Blood and Gastric Mucosa

ELISA kits were utilized to measure PLA_2_ and PGE_2_ level in blood and gastric mucosa. The test was performed in accordance with reagent instructions (R&D Systems, USA).

### 2.6. Western Blotting Analysis

The protein expression level of H^+^-K^+^-ATPase, COX-1, and COX-2 in gastric mucosal tissue was investigated by western blotting analysis. Each frozen stomach tissue was homogenized in RIPA buffer and centrifuged at 10,000 g for 15 min at 4°C. The protein concentration of the supernatant was measured using BCA protein assay kit (Beyotime Institute of Biotechnology, Shanghai, China) with bovine serum albumin as the standard sample. The protein (50 *μ*g/lane) was separated using SDS–PAGE and transferred to polyvinyl difluoride membrane (GE, Fairfield, CT, USA). The membrane was blocked with TBST containing 5% nonfat dry milk and incubated with anti-H^+^-K^+^-ATPase antibody (Abcam Biotechnology, USA, ab2866), anti-COX-1 antibody (Abcam Biotechnology, USA, ab133319), and anti-COX-2 antibody (Abcam Biotechnology, USA, ab52237) at a concentration of 1 : 2000 overnight at 4°C. The membrane was incubated with goat anti-mouse IgG (Abcam Biotechnology) and goat anti-rabbit IgG (Abcam Biotechnology) at a concentration of 1 : 5000 and subsequently visualized using an enhanced chemiluminescence (ECL) kit (Beyotime Biotechnology Company, Beijing, China). Equal lane loading was assessed using GAPDH.

### 2.7. Analysis of GTB Aqueous Extract Using UHPLC-Q-Exactive Hybrid Quadrupole-Orbitrap Mass

The GTB powder (0.01 g) from aqueous extract was dissolved in 80% methanol/distilled water (10 mL) with ultrasonic extraction at room temperature and centrifuged at 12,000 rpm for 10 min, respectively. After filtrated with 0.22 *μ*m filter membrane, the supernatant (1 *μ*L) was loaded into the UHPLC-MS system. Chromatographic separation was performed using Dionex Ultimate 3000 UHPLC system (Thermo Fisher Scientific, San Jose, CA, USA). The separation was achieved with Thermo Scientific Hypersil GOLD aQ C18 Column (2.1 mm × 100 mm, 1.9 *μ*m) at 40°C, and the flow rate was 0.4 mL/min. The mobile phase consisted of water containing acetonitrile (0.1% *v*/*v* formic acid) (A) and 0.1% *v*/*v* formic acid-H_2_O (B), which were applied in the gradient elution as follows: 5% A at 0-2 min, 5-95% A for 2-42 min, 95% A for 42-46.9 min, and 5% A for 47-50 min (the equilibration time was 3 min). A Q-exactive hybrid quadrupole-orbitrap mass spectrometer (Thermo Scientific, San Jose, CA, USA) included heat electrospray ionization (HESI) and was operated in both positive and negative ion modes to compete MS. The flow rate of sheath gas was 45 arbitrary units with the capillary temperature of 320°C. The auxiliary gas was set up to 15 arbitrary units at 350°C. In both positive and negative modes, the capillary voltage was set to +3.5 or -2.8 kV. The resolution of the full MS scan was 70,000 with the range of 80-1200 m*/*z. Samples were analyzed under 20, 30, and 40 normalized collision energy (NCE) in MS2 mode and resolution (17,500). Thermo Xcalibur 3.0 software (Thermo Scientific, San Jose, CA, USA) was used for collection and analysis of data.

### 2.8. Statistical Analysis

The results were expressed as means ± S.D. Differences between means were analyzed by one-way analysis of variance followed by Dunnett's or Student-Newman-Keuls test. Differences were considered statistically significant at *P* ≤ 0.05.

## 3. Results

### 3.1. The Effect of GTB Treatment on Gastric Acidity, Ulcer Index, and Volume of Gastric Juice

**Figure 1:**
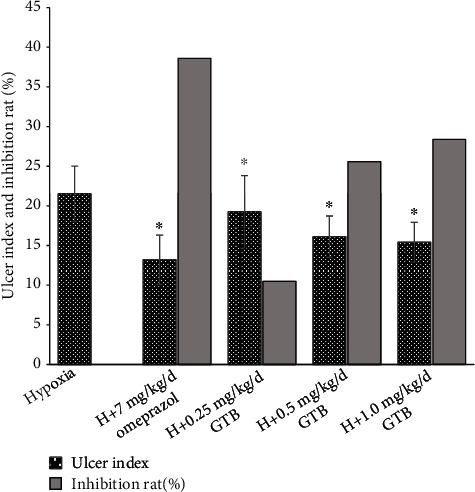
Effect of GTB on mean gross lesion index and inhibition rate of gastric mucosal ulcer in rat under acute systemic hypoxia for 6 days. The five groups including hypoxia (H), H+omeprazole, H+GTB 0.25 g/kg, H+GTB 0.5 g/kg, and H+GTB 1 g/kg were induced by systemic hypoxia (rats exposed to hypoxia in hypobaric chamber, equal to the parameter in altitude 5000 m) for 6 days. Each value represents the mean ± S.D. value of eight animals. ^∗^*P* < 0.05 vs. hypoxia group.

**Figure 2:**
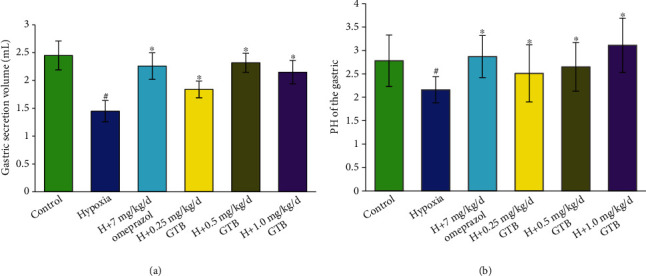
Effect of GTB on volume (a) and pH (b) of gastric juice in rat under acute systemic hypoxia for 6 days ($\bar{X}\pm s$, *n* = 12). Rats were exposed to hypoxia (in hypobaric chamber, equal to the parameter in altitude 5000 m), hypoxia (H)+omeprazole treatment (7 mg/kg/d), and hypoxia (H)+GTBs-treatment (0.25, 0.5, and 1.0 mg/kg/d) for 6 days. The volume and pH of the gastric juice were detected. Results are expressed as mean ± S.D.^#^*P* < 0.05 as compared with the control group; ^∗^*P* < 0.05 as compared with the hypoxia group.

We found that the gastric mucosal ulcer induced by systemic hypoxia was alleviated by GTB administration. Meanwhile, ulcer index was significantly increased under systemic hypoxia. After administrated by middle and high dosage of GTB and omeprazole, the ulcer index was significantly reduced (Figure 1). The volume of gastric juice was significantly reduced under systemic hypoxia and was significantly increased after GTB and omeprazole treatment. Compared with hypoxia group, gastric acidity was significantly reduced after treatment with middle and high dosages of GTB and omeprazole (Figure 2).

### 3.2. The Effect of GTB Treatment on Level of GAS, ET-1, and MTL

**Figure 3:**
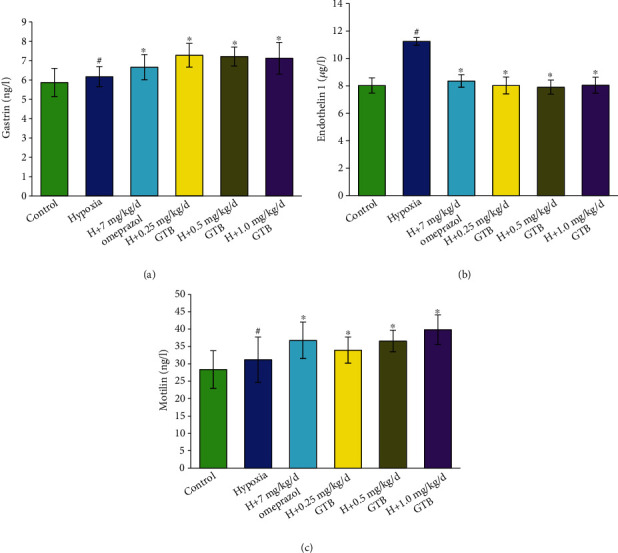
Effect of GTB on gastrin (a), endothelin-1 (b), and motilin (c) level in blood in rat under systemic hypoxia for 6 days ($\bar{X}\pm s$, *n* = 12). Rats were exposed to hypoxia (in hypobaric chamber, equal to the parameter in altitude 5000 m), hypoxia (H)+omeprazole treatment (7 mg/kg/d), and hypoxia (H)+GTBs-treatment (0.25, 0.5, and 1.0 mg/kg/d) for 6 days. The level of gastrin, endothelin-1, and motilin in blood in rat was detected by enzyme-linked immunosorbent assay (ELISA). Results were expressed as mean ± S.D.^#^*P* < 0.05 as compared with the control group; ^∗^*P* < 0.05 as compared with the hypoxia group.

GAS level was not obviously different among omeprazole and experimental groups. The level of ET-1 which was increased under hypoxia was significantly decreased after GTB and omeprazole treatment (*P* < 0.05). The MTL level had no significant difference between the hypoxia and control groups but was significantly increased after treatment with GTB (Figure 3).

### 3.3. The Effect of GTB Treatment on PLA_2_ and PGE_2_ Level in Serum and Gastric Mucosal Tissue

**Figure 4:**
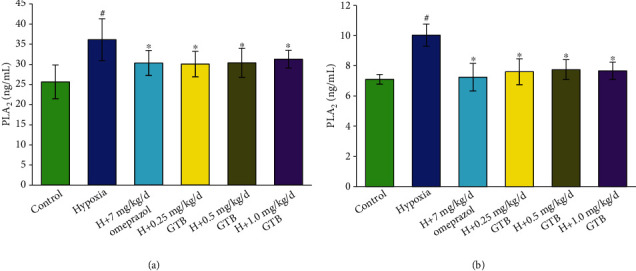
Effect of GTB on phospholipase A_2_ (PLA_2_) level in blood (a) and gastric mucosa (b) in rat under systemic hypoxia for 6 days ($\bar{X}\pm s$, *n* = 12). Rats were exposed to hypoxia (in hypobaric chamber, equal to the parameter in altitude 5000 m), hypoxia (H)+omeprazole treatment (7 mg/kg/d), and hypoxia (H)+GTBs-treatment (0.25, 0.5, and 1.0 mg/kg/d) for 6 days. The level of phospholipase A_2_ (PLA_2_) in blood and gastric mucosa in rat was detected by enzyme-linked immunosorbent assay (ELISA). Results were expressed as mean ± S.D.^#^*P* < 0.05 as compared with the control group; ^∗^*P* < 0.05 as compared with the hypoxia group.

**Figure 5:**
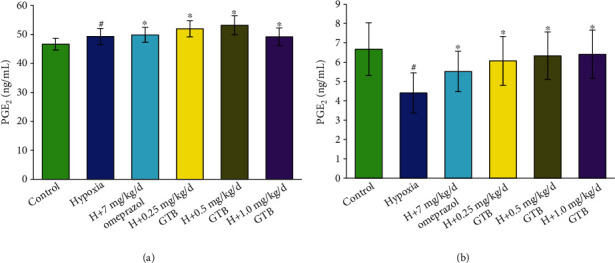
Effect of GTB on prostaglandin E_2_ (PGE_2_) level in blood (a) and gastric mucosa (b) in rat under systemic hypoxia for 6 days ($\bar{X}\pm s$, *n* = 12). Rats were exposed to hypoxia (in hypobaric chamber, equal to the parameter in altitude 5000 m), hypoxia (H)+omeprazole treatment (7 mg/kg/d), and hypoxia (H)+GTB-treatment (0.25, 0.5, and 1.0 mg/kg/d) for 6 days. The level of PGE_2_ in blood and gastric mucosa in rat was detected by enzyme-linked immunosorbent assay (ELISA). Results were expressed as mean ± S.D.^#^*P* < 0.05 as compared with the control group; ^∗^*P* < 0.05 as compared with the hypoxia group.

The level of PLA_2_ in serum and gastric mucosal tissue was significantly increased under systemic hypoxia and which was significantly decreased after GTB treatment (*P* < 0.05) (Figure 4). We found that the level of PGE_2_ which was decreased in gastric mucosal tissue was increased in serum under systemic hypoxia. After treatment with GTB, the level of PGE_2_ in serum and gastric mucosal tissue was both significantly increased compared with the hypoxia group (*P* < 0.05) (Figure 5).

### 3.4. The Effect of GTB Treatment on H^+^-K^+^-ATPase Protein Expression in Gastric Mucosal Tissue

**Figure 6:**
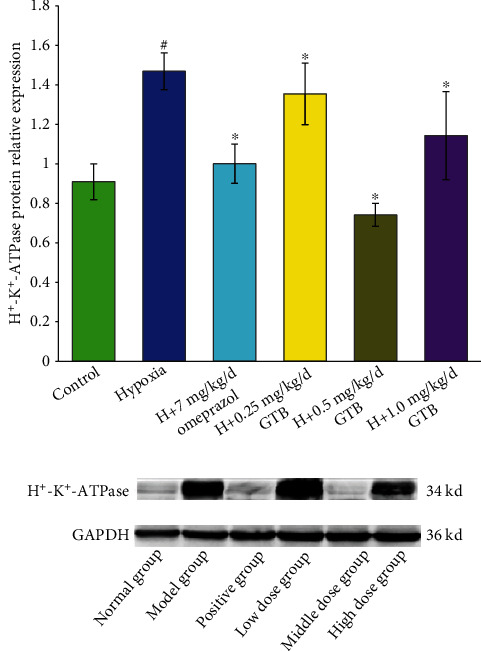
Effect of GTB on hydrogen potassium ATPase (H^+^-K^+^-ATPase) protein expression in gastric mucosal tissue detected by Western blotting. Rats were exposed to hypoxia (in hypobaric chamber, equal to the parameter in altitude 5000 m), hypoxia (H)+omeprazole treatment (7 mg/kg/d), and hypoxia (H)+GTBs-treatment (0.25, 0.5, and 1.0 mg/kg/d) for 6 days. GAPDH protein expression was used as a control. Relative expression levels of H^+^-K^+^-ATPase. Data were expressed as mean ± S.D. of three identical experiments. ^#^*P* < 0.05 as compared with the control group; ^∗^*P* < 0.05 as compared with the hypoxia group.

The protein expression level of H^+^-K^+^-ATPase was significantly increased under systemic hypoxia. Compared with the hypoxia group, the protein expression level of H^+^-K^+^-ATPase was downregulated after treatment with middle and high dosage of GTB and omeprazole (Figure 6).

### 3.5. The Effect of GTB Treatment on COX-1 and COX-2 Protein Expressions in Gastric Mucosal Tissue

**Figure 7:**
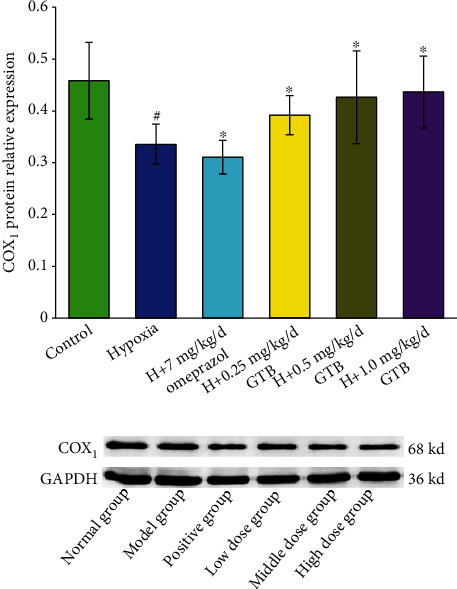
Effect of GTB on cyclooxygenase-1 (COX-1) protein expression in gastric mucosal tissue in rat detected by Western blotting. Rats were exposed to hypoxia (in hypobaric chamber, equal to the parameter in altitude 5000 m), hypoxia (H)+omeprazole treatment (7 mg/kg/d), and hypoxia (H)+GTBs-treatment (0.25, 0.5, and 1.0 mg/kg/d) for 6 days. GAPDH protein expression was used as a control. Relative expression levels of COX-1. Data are mean ± S.D. of three identical experiments. ^#^*P* < 0.05 as compared with the control group; ^∗^*P* < 0.05 as compared with the hypoxia group.

**Figure 8:**
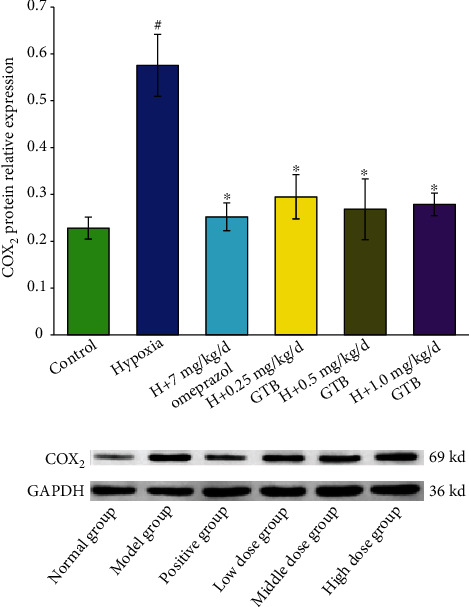
Effect of GTB on cyclooxygenase-2 (COX-2) protein expression in gastric mucosal tissue in rat detected by Western blotting. Rats were exposed to hypoxia (in hypobaric chamber, equal to the parameter in altitude 5000 m), hypoxia (H)+omeprazole treatment (7 mg/kg/d), and hypoxia (H)+GTBs-treatment (0.25, 0.5, and 1.0 mg/kg/d) for 6 days. GAPDH protein expression was used as a control. Relative expression levels of COX-2. Data are mean ± S.D. of three identical experiments. ^#^*P* < 0.05 as compared with the control group; ^∗^*P* < 0.05 as compared with the hypoxia group.

The COX-1 level was decreased significantly under systemic hypoxia. Middle and high dosages of GTB treatment upregulated COX-1 level in gastric *mucosal* tissue (Figure 7). The level of COX-2 was increased under systemic hypoxia which was downregulated by GTB administration (Figure 8).

### 3.6. Identification of the Compounds in GTB Using UHPLC-Q-Exactive Hybrid Quadrupole-Orbitrap Mass

**Figure 9:**
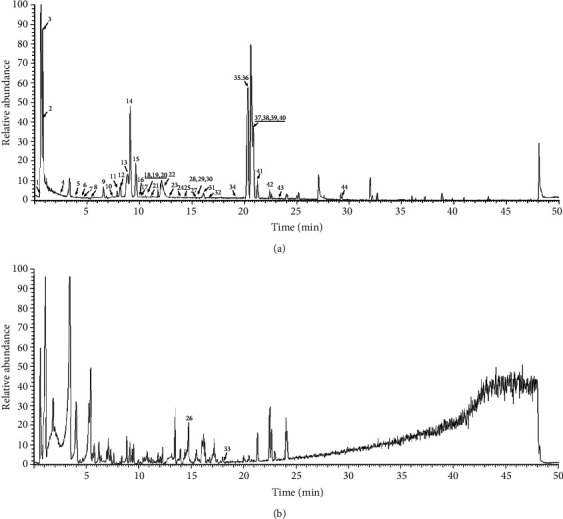
UHPLC-Q-exactive hybrid quadrupole-orbitrap mass analysis chromatogram of aqueous extract of GTB. (a) Total ion chromatograms (TIC) chromatogram in positive electrospray ionization (ESI) mode. (b) TIC chromatogram in negative ESI mode. Peaks 1–44 represent stachydrine, adenine, guanine, cinnamic acid, isovanillin, esculetin, 7,8-dihydroxycoumarin, anisic aldehyde, paeonol, corydine, boldine, phellodendrine, 7-hydroxycoumarin, bicuculline, protopine, berberrubine, baicalin, dihydropalmatine, allocryptopine, berberine, dehydroglaucine, dihydrosanguinarine, curcumol, micheliolide, diosmetin, andrographolide, isosteviol, carnosol, glabrolide, cafestol, quillaic acid, clareolide, 6-gingerol, piperine, atractylenolide II, isoalantolactone, dehydrocostus lactone, lindenenol, abietic acid, deoxyandrographolide, steviol, kahweol, nonivamide, and alpha-linolenic acid.

The total spectrum of chemical components in GTB aqueous extract was analyzed from both positive and negative ion models. 44 chemical components were identified by UHPLC-Q-Orbitrap MS analysis (Figure 9). It showed the characters of all 44 chemical constituents including chromatographic retention times, accurate molecular mass, and/or MS/MS data listed in Table 1. Among these, the peaks of 10, 11 12, 16, and 37 were identified as magnoflorine, boldine, phellodendrine, berberrubine, and dehydrocostus lactone, respectively, according to the data comparison with reference standards. Peak 10 was identified as magnoflorine with a protonated *m/z* 342.16998 ([M+H]^+^, C_20_H_24_NO_4_). The MS/MS experiment yielded a [M-(CH_3_)_2_NH]^+^ ion at *m/z* 297.11166 (C_18_H_17_O_4_) [16]. Peak 11 was protonated boldine *m/z* 328.15433 ([M+H]^+^, C_19_H_22_NO_4_). The MS/MS experiment yielded a [M-NH_2_CH_3_]^+^ ion at *m/z* 297.11176 (C_18_H_17_O_4_) [17]. Peak 12 was identified as phellodendrine with a protonated *m/z* 342.16998 (M^+^, C_20_H_24_NO_4_). The MS/MS experiment yielded a [M-C_9_H_10_O_2_-CH_3_]^+^ ion at *m/z* 177.07811 (C_10_H_11_NO_2_) [14]. Peak 16 was identified as berberrubine with protonated *m/z* 332.10738 ([M+H]^+^, C_19_H_16_NO_4_) [15]. Peak 37 was tentatively identified as dehydrocostus lactone with protonated *m/z* 231.13796 ([M+H]^+^, C_15_H_19_O_2_). The MS/MS experiment yielded a ion at *m/z* 185.13225 ([M-CO-H_2_O]^+^) [18]. Furthermore, other peaks were tentatively identified based on the chemical composition and MS/MS data and TCM database as well as previously published studies [16–18].

## 4. Discussion

Acute gastric mucosal lesion was life-threatening at high altitude where gastric mucosal balance may be disrupted. It was found that blood flow to the gastric mucosa decreased because of systemic hypoxia affecting the physiological balance between gastric acid secretion and gastric mucosal defense. Provided changes in the gastrointestinal tissue during hypoxia are explored, which should be intervened by medicines, especially by traditional medicines.

With a history going back approximately 2,500 years, Tibetan medicine is considered one of the world's oldest known traditional medicines [19]. Several traditional Tibetan medicines had been used to treat gastric diseases with obvious effect, and Grubthobrildkr is one of the classic Tibetan medicines to treat gastric problems. According to the reports, GTB attenuated acetic acid-induced gastric ulcer through reducing the expression of COX-2 and inflammatory reaction [20]. GTB also alleviated stress gastric ulcer induced by water immersion and pylorus ligature in rat [21]. Although GTB had been used for centuries as an effective and safe prescription for gastric disease treatment, its mechanism in the treatment of acute stress gastric ulcer under systemic hypoxia needs to be researched. In our previous study, we established systemic hypoxia-induced gastric ulcer rat model in 2, 4, 6, 8, and 10 days, respectively, and we observed that the severe gastric ulcer was in the 6-day hypoxia group [13]. The protective effect of GTB was also detected by hematoxylin and eosin staining and ultrastructural observation in systemic hypoxia-induced gastric ulcer rat model for 6 days [14]. In this article, we focused on detecting the gastrointestinal protective mechanism of GTB in rats.

The volume of gastric juice was significantly reduced, and total gastric acidity and ulcer index were significantly increased under systemic hypoxia in rat. The gastric ulcer index was reduced and pH of gastric juice and gastric secretion volume in rat were increased after GTB administration. GAS was from G cells of pyloric antrum for gastric acid secretion, and we found that GAS levels were not changed under systemic hypoxia for six days. MTL has been identified in the blood of dogs by means of radioimmunoassay [22], with function of stimulating pepsin output and enhancing activity of the stomach [23]. We found that MTL levels were not influenced by systemic hypoxia for six days in rats. ET-1 was one of the proinflammatory cytokines for the contraction of blood vessels, playing an important role in gastric ulcer formation. The increasing secretion of ET-1 results in the occurrence of hypoxia, acidosis, and ulcers. We found that ET-1 level was increased under hypoxia. GTB administration decreased ET-1 level and increased the level of MTL in the blood significantly compared with the hypoxia group but GAS level was not influenced. The results could be explained that the effects of GTB increasing pH of gastric juice and gastric secretion volume were mainly regulated by ET-1 and MTL levels. GAS, MTL, and ET-1 were all found to influence the level of intercellular Ca^2+^ and eventually activated H^+^-K^+^-ATPase [24]. H^+^-K^+^-ATPase are responsible for secreting acid into the gastric lumen, which catalyzes the exchange of one H^+^ for one K^+^ at the expense of an ATP molecule [25]. We found that the H^+^-K^+^-ATPase level in gastric mucosal tissue was increased under systemic hypoxia. GTB reversed the increased protein expression of H^+^-K^+^-ATPase in gastric mucosal tissue induced by systemic hypoxia.

Prostaglandins (PGs), targets for the prophylactic effect of probiotics in gastric ulcers [23], were participant in the ulcer healing process by decreasing acid secretion, stimulating the production of mucus, bicarbonate, and phospholipids [26]. Enzymes involved in PGs synthesis include PLA_2_, which influenced the production of arachidonic acid, COX-1, and COX-2. PGE_2_ is a member of PGs, the restoration of which can reduce gastric mucosa lesions [8, 9]. GTB treatment reduced PLA_2_ level both in serum and in gastric tissue in rat under systemic hypoxia. Although the level of PGE_2_ in serum was increased in the six-day hypoxia group, GTB treatment increased PGE_2_ level both in the serum and in gastric tissue. Based on the dual contribution of PGs to inflammation and mucosal defense, the increased PGE_2_ level after GTB administration could be deduced to play a protective role in ulcer lesions under systemic hypoxia.

COX-1 was a house-keeping enzyme that produces cytoprotective PGs, while COX-2 was an inducible form of the enzyme that produces inflammatory PGs. The protein expression of COX-1 was found to be reduced, but COX-2 was increased under acute systemic hypoxia. GTB treatment was found to increase the protein expression level of COX-1 and decrease that of COX-2 in gastric tissue in rat. 44 constituents in Grubthobrildkr were identified by UHPLC-Q-Orbitrap MS. To the best of our knowledge, we did not find articles which report the relationship between the 44 ingredients and the treatment of gastric ulcer.

## 5. Conclusion

Traditional Tibetan patent medicine Grubthobrildkr showed a protective effect and alleviated the ulceration in gastric mucosa under systemic hypoxia. The effect of GTB increasing volume and pH of gastric juice in rat under acute systemic hypoxia could be regulated by MTL and ET-1. The molecular mechanism of GTB might be related to reduction of H+-K+-ATPase protein expression and regulation of prostaglandin family by downregulating COX-2 expression and upregulating COX-1 protein expression in the gastric mucosa of rats under systemic hypoxia. 44 constituents in GTB were identified by UHPLC-Q-TOF-MS/MS. Furthermore, comprehensive studies are needed to elucidate the gastroprotective mechanism of GTB.

## Figures and Tables

**Table 1:** Compounds identified in aqueous extract of GTB by UHPLC-Q-exactive hybrid quadrupole-orbitrap mass analysis.

No.	t_R_ (min)	MS (*m/z*)			MS/MS (*m/z*)	Molecular formula	Identification
		Observed mass (Da)	Calculated mass (Da)	Error (ppm)			
1	0.64	144.10191 [M+H]^+^	144.10175	-1.11	103.13138, 84.08134, 98.09679, and 70.06587	C_7_H_13_NO_2_	Stachydrine
2	0.73	136.06177 [M+H]^+^	136.06165	-0.88	109.05100	C_5_H_5_N_5_	Adenine
3	0.86	152.05669 [M+H]^+^	152.05661	-0.53	110.03516, 135.02991, 128.04541, and 107.04948	C_5_H_5_N_5_O	Guanine
4	2.5	149.05971 [M+H]^+^	149.05962	-0.60	121.06486, 118.04142, 131.04907, and 103.05457	C_9_H_8_O_2_	Cinnamic acid
5	3.82	153.05462 [M+H]^+^	153.05449	-0.85	125.05972, 93.07038, 111.96861, and 129.97884	C_8_H_8_O_3_	Isovanillin
6	4.39	179.03389 [M+H]^+^	179.03365	-1.34	151.03896,114.94835, 123.04412, and 133.02834	C_9_H_6_O_4_	Esculetin
7	4.4	179.03389 [M+H]^+^	179.03365	-1.34	123.04412, 117.03340	C_9_H_6_O_4_	7,8-Dihydroxycoumarin
8	5.36	137.05971 [M+H]^+^	137.05962	-0.66	109.06524	C_8_H_8_O_2_	Anisic aldehyde
9	6.63	167.07027 [M+H]^+^	167.07007	-1.20	125.05970, 121.10149, 84.96030, and 110.03656	C_9_H_10_O_3_	Paeonol
10	7.26	342.16998 [M+H]^+^	342.16922	-2.22	297.11166, 265.08533, and 237.09053	C_20_H_23_NO_4_	Magnoflorine
11	7.92	328.15433 [M+H]^+^	328.15372	-1.86	237.09117, 297.11176, 178.08595, and 163.06247	C_19_H_21_NO_4_	Boldine
12	8.28	342.16998 M^+^	342.16934	-1.87	192.10162, 177.07811	C_20_H_24_NO_4_	Phellodendrine
13	8.84	163.03897 [M+H]^+^	163.03870	-1.66	107.04945	C_9_H_6_O_3_	7-Hydroxycoumarin
14	9.15	368.11286 [M+H]^+^	368.11218	-1.85	307.05954, 277.04910, 249.05411, and 190.08597	C_20_H_17_NO_6_	(+)Bicuculline
15	9.73	354.13360 [M+H]^+^	354.13281	-2.23	188.07033, 275.06979, 188.07033, and 149.05962	C_20_H_19_NO_5_	Protopine
16	9.86	322.10738 [M+H]^+^	322.10690	-1.49	279.08868, 234.09065, 307.08350, and 250.08571	C_19_H_15_NO_4_	Berberrubine
17	9.95	447.09219 [M+H]^+^	447.09177	-0.94	—	C_21_H_18_O_11_	Baicalin
18	10.39	354.16998 [M+H]^+^	354.13232	-106.33	336.12201, 320.09183, 190.08597, and 275.06976	C_21_H_23_NO_4_	Dihydropalmatine
19	10.60	370.16490 [M+H]^+^	370.16428	-1.67	290.09338, 188.07036	C_21_H_23_NO_5_	Allocryptopine
20	10.96	336.12303 M^+^	336.12247	-1.67	278.08099, 292.09616	C_20_H_18_NO_4_	Berberine
21	11.17	354.16998 [M+H]^+^	354.16943	-1.55	338.13928, 306.12180, 192.10165, and 165.09084	C_21_H_23_NO_4_	Dehydroglaucine
22	12.22	334.10738 [M+H]^+^	334.10669	-2.07	319.08340, 261.07614, 302.07990, and 290.08054	C_20_H_15_NO_4_	Dihydrosanguinarine
23	12.78	237.18491 [M+H]^+^	237.18472	-0.80	196.01671, 182.98506	C_15_H_24_O_2_	Curcumol
24	14.00	249.14852 [M+H]^+^	249.14781	-2.85	231.13780, 185.13234, 135.08034, and 119.08567	C_15_H_20_O_3_	Micheliolide
25	14.24	301.07066 [M+H]^+^	301.07016	-1.66	286.04666, 147.11650, 258.05185, and 229.04871	C_16_H_12_O_6_	Diosmetin
26	14.72	351.2166 [M+H]^−^	351.21790	3.70	333.20121, 305.21259, 289.21765, and 183.10057	C_20_H_30_O_5_	Andrographolide
27	15.52	319.22677 [M+H]^+^	319.22577	-3.13	273.22053, 255.21010, 301.21591, and 147.11681	C_20_H_30_O_3_	Isosteviol
28	16.00	331.19039 [M+H]^+^	331.18976	-1.90	285.18408, 215.10663, 203.10638, and 171.08023	C_20_H_26_O_4_	Carnosol
29	16.12	469.33075 [M+H]^+^	469.33124	1.04	95.08595, 299.20111, 119.08565, and 405.31430	C_30_H_44_O_4_	Glabrolide
30	16.14	317.21112 [M+H]^+^	317.21118	0.19	281.19016, 131.08543, 299.20016, and 271.20538	C_20_H_28_O_3_	Cafestol
31	16.16	487.34024 [M+H]^+^	487.34180	3.20	451.32074, 187.14790, 119.08562, and 201.16367	C_30_H_46_O_5_	Quillaic acid
32	16.66	251.20056 [M+H]^+^	251.20020	-1.43	1187.14793, 215.17897, 233.18958, and 95.08595	C_16_H_26_O_2_	Clareolide
33	18.00	293.17583 [M+H]^−^	293.17603	0.68	236.10522, 177.09090, 221.15428, and 249.18590	C_17_H_26_O_4_	6-Gingerol
34	19.08	286.14377 [M+H]^+^	286.14334	-1.50	201.05440, 143.04912,135.04396, and 115.05444	C_17_H_19_NO_3_	Piperine
35	20.38	233.15361 [M+H]^+^	233.15335	-1.12	187.14793, 145.10107	C_15_H_20_O_2_	Atractylenolide II
36	20.28	233.15361 [M+H]^+^	233.15334	-1.16	187.14796, 159.11668, 215.14293, and 145.10109	C_15_H_20_O_2_	Isoalantolactone
37	20.84	231.13796 [M+H]^+^	231.13757	-1.69	185.13225, 143.08539, 195.11664, and 157.10092	C_15_H_18_O_2_	Dehydrocostus lactone
38	20.84	231.13796 [M+H]^+^	231.13757	-1.69	105.07019, 98.03719, 119.08562, and 131.08542	C_15_H_18_O_2_	Lindenenol
39	21.08	303.23186 [M+H]^+^	303.23138	-1.58	257.22589, 123.12687, and 147.11668	C_20_H_30_O_2_	Abietic acid
40	21.22	335.22169 [M+H]^+^	335.22305	4.06	289.21722, 129.90915, and 275.20213	C_20_H_30_O_4_	Deoxyandrographolide
41	21.27	319.22677 [M+H]^+^	319.22650	-0.85	227.14243, 273.22098, 255.21030, and 161.13228	C_20_H_30_O_3_	Steviol
42	22.42	315.19547 [M+H]^+^	315.19513	-1.08	303.97168, 145.06465, 187.11153, and 269.18951	C_20_H_26_O3	Kahweol
43	23.26	294.20637 [M+H]^+^	294.20685	1.63	161.09586, 137.05959, 179.10640, and 203.10635	C_17_H_27_NO_3_	Nonivamide
44	29.25	279.23186 [M+H]^+^	279.23145	-1.47	95.08595, 81.07049, and 67.05501	C_18_H_30_O_2_	Alpha-linolenic acid

t_R_: retention time.

## Data Availability

The data used to support the findings of this study are included within the article.

## Glossary

GTB: Grubthobrildkr
ET-1: Endothelin-1
GAS: Gastrin
PLA_2_: Phospholipase A_2_
PGE_2_: Prostaglandin E_2_
H^+^-K^+^-ATPase: Hydrogen potassium ATPase
COX-1: Cyclooxygenase-1
COX-2: Cyclooxygenase-2
UHPLC-Q-Orbitrap MS: UHPLC-Q-exactive hybrid quadrupole-orbitrap mass.

## Additional Points

*Article Info*. Chemical compounds studied in this article: magnoflorine (Pubchem CID: 73337), boldine (Pubchem CID: 10154), phellodendrine (Pubchem CID: 59819), berberrubine (Pubchem CID: 72703), and dehydrocostus lactone (Pubchem CID: 73174).

## References

[1] Wu T. Y., Ding S. Q., Liu J. L. (2007). High-altitude gastrointestinal bleeding: an observation in Qinghai-Tibetan railroad construction workers on Mountain Tanggula. *World Journal of Gastroenterology*.

[2] Marik P. E., Vasu T., Hirani A., Pachinburavan M. (2010). Stress ulcer prophylaxis in the new millennium: a systematic review and meta-analysis. *Critical Care Medicine*.

[3] Syam A. F., Simadibrata M., Wanandi S. I., Hernowo B. S., Sadikin M., Rani A. A. (2011). Gastric ulcers induced by systemic hypoxia. *Acta Medica Indonesiana*.

[4] Michida T., Kawano S., Masuda E. (1997). Endothelin-1 in the gastric mucosa in stress ulcers of critically ill patients. *The American Journal of Gastroenterology*.

[5] Lundell L. (2015). The physiological background behind and course of development of the first proton pump inhibitor. *Scandinavian Journal of Gastroenterology*.

[6] Ribeiro A. R., Diniz P. B., Pinheiro M. S., Albuquerque-Júnior R. L., Thomazzi S. M. (2016). Gastroprotective effects of thymol on acute and chronic ulcers in rats: the role of prostaglandins, ATP-sensitive K(+) channels, and gastric mucus secretion. *Chemico-Biological Interactions*.

[7] Harada S., Takeuchi T., Edogawa S., Ota K., Kojima Y., Higuchi K. (2015). The availability of prostaglandin derivatives in a treatment and prevention for gastrointestinal mucosal injury. *Nihon Rinsho*.

[8] Tanaka A., Hatazawa R., Takahira Y., Izumi N., Filaretova L., Takeuchi K. (2007). Preconditioning stress prevents cold restraint stress-induced gastric lesions in rats: roles of COX-1, COX-2, and PLA2. *Digestive Diseases and Sciences*.

[9] Yamamoto S., Ohki S., Ogino N. (1980). Enzymes involved in the formation and further transformations of prostaglandin endoperoxides. *Advances in Prostaglandin and Thromboxane Research*.

[10] Meier R., Hengstler P., Weber F., Maurer H., Bommeli C., Brignoli R. (2013). The Tibetan herbal formula Padma Digestin in functional dyspepsia: an open-label study. *Forschende Komplementärmedizin*.

[11] Chen Y. P., Wang S. Y., Lu H. Y., Chen C. J., Li X. J. (2008). Clinical observation of Tibetan medicine Grubthobrildkr in peptic ulcer. *J. Qiqihai. Univer. Med*.

[12] Wang Y., Song Z., Chen Y. P. (2010). Clinical observation of Tibetan medicine Grubthobrildkr on peptic ulcer. *Chin. J. Trauma. Disability*.

[13] LI Y., Yang M. (2017). Establishment of rat model of plateau hypoxia caused stress ulcer. *Chongqing Medicine*.

[14] Yang M., Deng L. Z., Li Z. Q., Li Y. F., Lu D. X. (2008). The changes of gastrin and motilin in exposed to hypobaric hypoxia and the effect of Tibetan medicine in rats. *Journal of Qinghai Medical College*.

[15] Dekanski D., Janićijević-Hudomal S., Ristić S. (2009). Attenuation of cold restraint stress-induced gastric lesions by an olive leaf extract. *General Physiology and Biophysics*.

[16] Chen Y., Zhang Z., Zhang Y. (2019). A new method for simultaneous determination of phenolic acids, alkaloids and limonoids in Phellodendri Amurensis Cortex. *Molecules*.

[17] Wu Y. J., Zheng Y. L., Luan L. J. (2010). Development of the fingerprint for the quality of Radix Linderae through ultra-pressure liquid chromatography-photodiode array detection/electrospray ionization mass spectrometry. *Journal of Separation Science*.

[18] He Y., Cheng P., Wang W. (2018). Rapid investigation and screening of bioactive components in simo decoction via LC-Q-TOF-MS and UF-HPLC-MD methods. *Molecules*.

[19] Roberti di Sarsina P., Ottaviani L., Mella J. (2011). Tibetan medicine: a unique heritage of person-centered medicine. *The EPMA Journal*.

[20] Zhao Y., Luo C., Lan X. M., Liu Y., Sun Y. Z. (2016). Mechanism of Zhituo Jiebai pills on gastric ulcer induced by acetic acid in mice. *J. JiangXi. Univ. TCM*.

[21] Liu Y., Lan X. M., Luo C., Zhao Y. (2016). Study on the effect of Zhituo Jiebai pill on gastric ulcer in rats. *Lishizhen Med. Mater. Med. Res*.

[22] Itoh Z., Takeuchi S., Aizawa I. (1978). Changes in plasma motilin concentration and gastrointestinal contractile activity in conscious dogs. *Journal of Digestive Diseases*.

[23] Khoder G., Al-Menhali A. A., Al-Yassir F., Karam S. M. (2016). Potential role of probiotics in the management of gastric ulcer. *Experimental and Therapeutic Medicine*.

[24] Schubert M. L. (2014). Gastric secretion. *Current Opinion in Gastroenterology*.

[25] Chung C. S., Chiang T. H., Lee Y. C. (2015). A systematic approach for the diagnosis and treatment of idiopathic peptic ulcers. *The Korean Journal of Internal Medicine*.

[26] Hatazawa R., Ohno R., Tanigami M., Tanaka A., Takeuchi K. (2004). Roles of endogenous prostaglandins and cyclo-oxygenase izoenzymes in mucosal defense of inflamed rat stomach. *Journal of Pharmacology and Experimental Therapeutics*.

